# Staphylococcal Superantigens Stimulate Epithelial Cells through CD40 To Produce Chemokines

**DOI:** 10.1128/mBio.00214-19

**Published:** 2019-03-19

**Authors:** Patrick M. Schlievert, Michael P. Cahill, Bruce S. Hostager, Amanda J. Brosnahan, Aloysius J. Klingelhutz, Francoise A. Gourronc, Gail A. Bishop, Donald Y. M. Leung

**Affiliations:** a Department of Microbiology and Immunology, Carver College of Medicine, University of Iowa, Iowa City, Iowa, USA; b VAMC, Iowa City, Iowa, USA; c Department of Microbiology and Immunology, School of Medicine, University of Minnesota, Minneapolis, Minnesota, USA; d Department of Pediatrics, National Jewish Health, Denver, Colorado, USA; University of Wisconsin-Madison; University of Idaho; Mississippi State University

**Keywords:** CD40, *Staphylococcus aureus*, chemokines, superantigens, toxic shock syndrome toxin

## Abstract

Mucosal and skin tissues form barriers to infection by most bacterial pathogens. Staphylococcus aureus causes diseases across these barriers in part dependent on the proinflammatory properties of superantigens. We showed, through use of a CRISPR-Cas9 CD40 knockout, that the superantigens toxic shock syndrome toxin 1 (TSST-1) and staphylococcal enterotoxins (SEs) B and C stimulated chemokine production from human vaginal epithelial cells (HVECs) through human CD40. This response was enhanced by addition of antibodies against CD40 through an unknown mechanism. TSST-1 was better able to stimulate chemokine (IL-8 and MIP-3α) production by HVECs than SEB and SEC, suggesting this is the reason for TSST-1’s exclusive association with menstrual TSS. A mutant of TSST-1, K121A, caused TSS in a rabbit model when administered vaginally but not intravenously, emphasizing the importance of the local vaginal environment. Collectively, our data suggested that superantigens facilitate infections by disruption of mucosal barriers through their binding to CD40, with subsequent expression of chemokines. The chemokines facilitate TSS and possibly other epithelial conditions after attraction of the adaptive immune system to the local environment.

## Abstract (precis)

Menstrual toxic shock syndrome (TSS) is a serious infectious disease associated with vaginal colonization by Staphylococcus aureus producing the exotoxin TSS toxin 1 (TSST-1). We show that menstrual TSS occurs after TSST-1 interaction with an immune costimulatory molecule called CD40 on the surface of vaginal epithelial cells. Other related toxins, where the entire family is called the superantigen family, bind to CD40, but not with a high-enough apparent affinity to cause TSS; thus, TSST-1 is the only exotoxin superantigen associated. Once the epithelial cells become activated by TSST-1, they produce soluble molecules referred to as chemokines, which in turn facilitate TSST-1 activation of T lymphocytes and macrophages to cause the symptoms of TSS. Identification of small-molecule inhibitors of the interaction of TSST-1 with CD40 may be useful so that they may serve as additives to medical devices, such as tampons and menstrual cups, to reduce the incidence of menstrual TSS.

## INTRODUCTION

Superantigens (SAgs) are a large family of proteins produced by staphylococci, primarily Staphylococcus aureus, and beta-hemolytic streptococci (1–3). There are at least 22 S. aureus SAgs, designated staphylococcal enterotoxins (SEs) A to E and G, staphylococcal enterotoxin-like (SE*l*) H to X, and toxic shock syndrome toxin 1 (TSST-1). The subfamilies within the staphylococcal SAgs have unique activities (for example, the SEs cause food poisoning and emesis) (1–3). However, all SAgs share the properties of binding to the variable component of the β-chain of T cell receptors (Vβ-TCRs) and the α- and/or β-chains of major histocompatibility complex (MHC) II molecules (1, 2, 4). The result of these interactions is massive T lymphocyte activation and proliferation plus activation of macrophages to produce proinflammatory cytokines, including but not limited to tumor necrosis factors α and β, interleukin-1β, interleukin-2, and gamma interferon (1, 2, 5).

While the interactions of SAgs with Vβ-TCRs and MHC II have been extensively studied, other receptors for SAgs have been proposed but remain relatively unstudied. For example, there is a dodecapeptide region, relatively highly conserved in SAgs, that has been proposed to bind to alternative receptors on immune cells (CD28), epithelial cells (possibly CD40), keratinocytes, and adipocytes (gp130) (6–13). Minor changes in the amino acid sequence of the dodecapeptide region may explain the greater mucosal penetration of TSST-1 than of other SAgs (1, 11).

The majority of S. aureus infections are initiated from mucosal surfaces, where epithelial cells dominate those barriers. It is well known that TSST-1 causes 100% of menstrual TSS, and in the vast majority of instances the causative USA200 strain of S. aureus remains on the mucosal surfaces (1, 14, 15). Nonmenstrual staphylococcal TSS primarily is caused by TSST-1 (50%) and SEs B and C (50%) (1–3); SEB and SEC are 75% identical and have nearly identical contact amino acid residues with Vβ-TCRs and MHC II molecules (1, 2, 16, 17). Our prior studies suggest that epithelial cells may be important in initiation of harmful inflammatory responses to disrupt the mucosal barrier and facilitate onset of TSS (18–21).

We previously showed that highly purified TSST-1 interacts with isolated human vaginal epithelial cells (HVECs) to upregulate over 500 genes (18). These cells have important typical characteristics, including that they lack Toll-like receptor-4 (TLR4) and thus are not responsive to lipopolysaccharide (LPS); the cells do have TLR2/6 on their surfaces. As expected, the HVEC line also forms partial tight junctions, with barrier function dependent on layers of cells and lipid vesicles (18, 22, 23). HVECs also characteristically lack MHC II molecules, but they can be stimulated with gamma interferon to upregulate MHC II expression. Although constitutively present on the surface of HVECs, when treated with TSST-1, one of the upregulated genes in HVECs encodes CD40 (18). CD40 can be expressed by a variety of cell types, but it is particularly important for the normal activation of the antigen-presenting cells of the immune system, such as B cells, macrophages, and dendritic cells. CD40 exists as either monomers or preformed trimers that assemble to form a complex that interacts with the CD40 ligand, CD154, found on activated CD4 T cells and platelets (24). However, the role of CD40 on HVECs, if any, in inflammation or adaptive immune responses to SAgs remains unknown.

We have recently shown that humans with diabetes mellitus type II have large numbers of S. aureus organisms on their surfaces (9). These strains produce significant amounts of the SAgs TSST-1, SEB, and SEC (9). We have used the amounts of SAgs found on human surfaces to produce diabetes mellitus II in a rabbit model in which the SAg is applied in subcutaneously implanted miniosmotic pumps (9). We hypothesized that the SAgs may be interacting with multiple human cells in a way similar to HVECs to facilitate chronic inflammation with consequent development of diabetes mellitus II.

The goal of this study was to characterize more fully the role of S. aureus SAgs in stimulating HVECs as representative of mucosal epithelial cells, focusing on the role of CD40 in this interaction. We hypothesized that SAg interaction with CD40 on such cells is beneficial to the pathogen through signaling by induced chemokines to disrupt the mechanical barriers and facilitating inflammatory responses and TSS.

## RESULTS

Prior research suggests that many pathogens cause human diseases across mucosal surfaces at least in part through induction of chemokine (for example, IL-8 and MIP-3α) production by epithelial cells, such as human vaginal epithelial cells (HVECs) (20, 25, 26). TSST-1 induces chemokine production at these barriers, thereby recruiting innate and adaptive immune cells, some of which participate in barrier disruption through inflammation (18, 20, 22, 23). Subsequently, there is massive activation of CD4 T cells and macrophages to cause a cytokine storm that we see as TSS (1). We initiated studies to assess the local vaginal mucosal environment in production of TSS and possibly other conditions.

### Shared dodecapeptide region of superantigens (SAgs).

**FIG 1:**
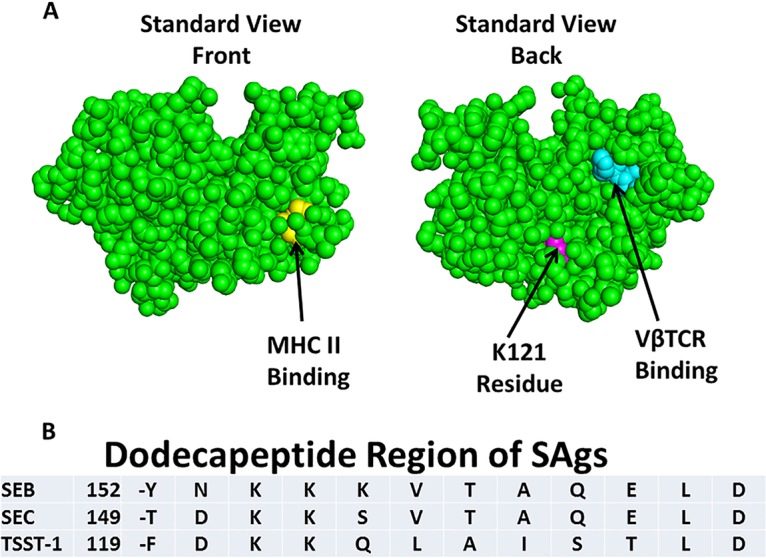
Structure as determined by PyMOL (RCSB Protein Data Bank 3TSS) of TSST-1 according to the standard front and rear views (A) and amino acid residues comprising the dodecapeptide region of SAgs (B). Residues involved in MHC II binding are shown in yellow (not all contact residues included), residues involved in Vβ2-TCR binding are shown in cyan (does not include all residues), and the location of residue Lys 121 is in magenta. The dodecapeptide regions for SEB, SEC, and TSST-1 are shown with a number representing the amino acid residue immediately preceding the dodecapeptide.

**FIG 2:**
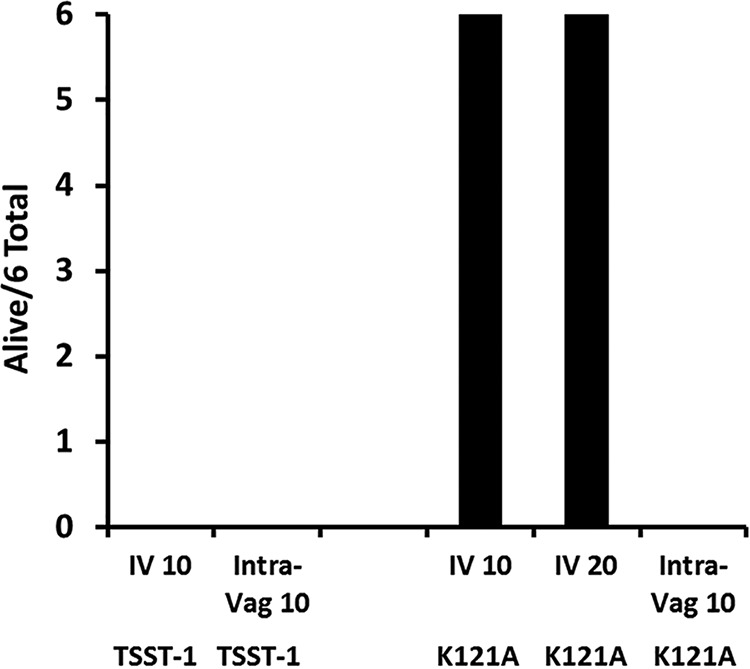
Lethality of TSST-1 and a K121A mutant of TSST-1, where Lys at position 121 was changed to Ala, as tested in two TSS models. The number of rabbits alive out of a total of 6 is shown after i.v. (10 or 20 µg/kg) or intravaginal (Intra-Vag; 10 µg/kg) administration. By Fisher’s exact probability test, *P* < 0.001 for all comparisons to rabbits administered the K121A mutant i.v.

Multiple studies suggest that a shared dodecapeptide region of SAgs contributes to biological activities, notably epithelial barrier penetration and immune cell activation (1, 6, 7, 19). Figure 1A shows the three-dimensional structure of TSST-1, with an important amino acid residue of the TSST-1 dodecapeptide highlighted (Lys 121, magenta). The figure also shows two key amino acids (Gly 31 and Ser 32, yellow) in the MHC II binding domain and His 135 and Gln 136 (cyan) in the Vβ2-TCR binding domain. The single-amino-acid designations for the entire dodecapeptide region of TSST-1, SEB, and SEC are shown in Fig. 1B. The dodecapeptide region of TSST-1 has the greatest divergence from this same structural region in other SAgs. It has been hypothesized that this divergence accounts for TSST-1’s greater penetration of the vaginal mucosal barrier compared to other SAgs and thus unique association with menstrual TSS (8, 11, 12). While studying this 12-amino-acid region of TSST-1, we tested various single amino acid changes through alanine scanning for altered ability to cause TSS in rabbits using two models, one intravenous (i.v.) (systemic) and one intravaginal (local). One mutant was distinct from the others, that of K121A, where the Lys at position 121 was changed to Ala. K121 is partially surface exposed in TSST-1 (Fig. 1A). Upon i.v. (systemic) or vaginal (local) administration, wild-type TSST-1 (10 µg/kg of body weight) was 100% lethal by 48 h postinjection (Fig. 2) in the two TSS models, with 6/6 animals succumbing in both groups. However, at two separate doses (10 μg/kg and 20 μg/kg), the K121A TSST-1 mutant was not lethal when administered i.v. (systemically), with 0/6 animals succumbing in each group. Surprisingly, the K121A mutant was lethal when administered to rabbits intravaginally. These data, while at first difficult to understand, suggest that the K121A TSST-1 mutant, as administered intravaginally (locally), had the ability to interact with a host cell receptor in the vagina, facilitating submucosal TSST-1 transit and activation of locally resident CD4 T cells and macrophages, to result in a cytokine storm manifested as TSS.

### SAgs cause production of proinflammatory chemokines from human vaginal epithelial cells (HVECs).

**FIG 3:**
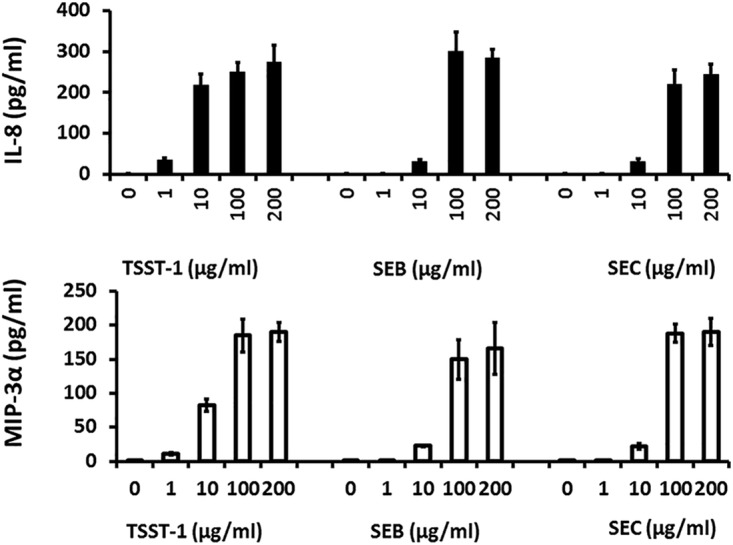
Chemokine (IL-8 and MIP-3α) production by human vaginal epithelial cells after 6 h of incubation in response to various concentrations of the SAgs TSST-1, SEB, and SEC. Bars indicate SD. Chemokines quantified by ELISA. At 1 and 10 µg/ml, TSST-1 induced greater chemokine production than SEs B and C (*P* < 0.001).

The most likely first cell types encountered by TSST-1, as secreted by S. aureus colonizing the vaginal surface, are epithelial cells. A dose-response study was thus performed with use of TSST-1, SEB, or SEC to assess chemokine (IL-8 and MIP-3α) production by HVECs (Fig. 3). TSST-1 induced significant production of both chemokines from 1 to 200 µg/ml. In contrast, SEs B and C induced chemokines at SAg concentrations of 10 to 200 µg/ml. At 100 and 200 µg/ml, there was no significant difference in chemokine induction by all three SAgs. Overall, TSST-1 was 10 to 50 times more active in inducing chemokine production by HVECs.

In all following studies, we most often used SAgs at 100 µg/ml since this dose reliably caused significant chemokine production by all three SAgs. We have shown in prior studies that S. aureus strains have the ability to secrete up to 16,000 to 20,000 µg/ml of TSST-1, SEB, and SEC in biofilm cultures as would be expected on the vaginal mucosa with tampon use (27). Thus, the 100-µg/ml dose chosen for further studies was physiologic. Additionally, for the majority of studies we assumed that SEB and SEC would function similarly because of their highly shared structures; we thus used TSST-1 and SEB for subsequent assays.

### SAgs plus antibodies against CD40 show enhanced chemokine production.

**FIG 4:**
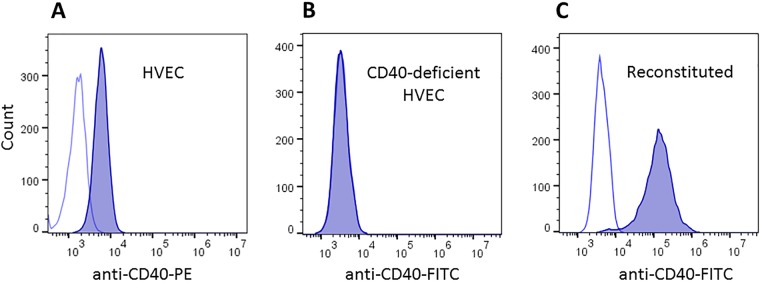
Flow cytometry of (A) human vaginal epithelial cells (HVECs) stained with antibodies to CD40 (phycoerythrin [PE] labeled) or an isotype-matched irrelevant antibody, (B) CD40-deficient HVECs through CRISPR-Cas9 knockout stained with FITC-labeled antibodies to CD40, and (C) deficient HVECs reconstituted with CD40 on a plasmid as stained with FITC-labeled antibodies or isotype-matched irrelevant antibodies.

Our prior human microarray studies demonstrate that, among other molecules, expression of CD40 is upregulated in response to TSST-1 challenge. TSST-1 binds to CD40 with a *K_d_* of 2.7 × 10^−6^ M (28). We were previously unable to demonstrate SEB or SEC binding to CD40, possibly because of low or no affinity of these two SAgs for the molecule. Our studies showed that nonstimulated HVECs have CD40 on their surfaces (Fig. 4A).

**FIG 5:**
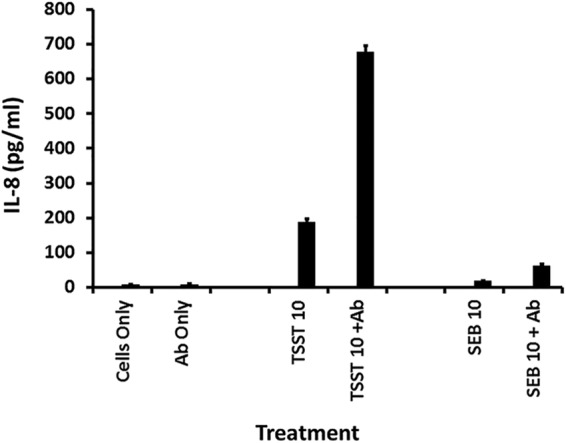
Chemokine (IL-8) production by human vaginal epithelial cells after 6-h exposure to TSST-1 (10 µg), TSST-1 (10 µg plus 20 µl of antibodies [Ab] against CD40), SEB (10 µg), SEB (10 µg plus 20 µl of antibodies against CD40), 20 µl of antibodies alone, or cells alone in keratinocyte serum-free medium (KSFM). Bars indicate SD. All responses in the presence of SAg with or without antibodies are significantly greater than HVECs in KSFM alone or KSFM plus antibodies (*P* < 0.001). Additionally, the responses of SAg alone compared to SAg plus antibodies were significantly different (*P* < 0.001). Finally, responses to TSST-1 with or without antibodies were greater than SEB with or without antibodies (*P* < 0.001).

**FIG 6:**
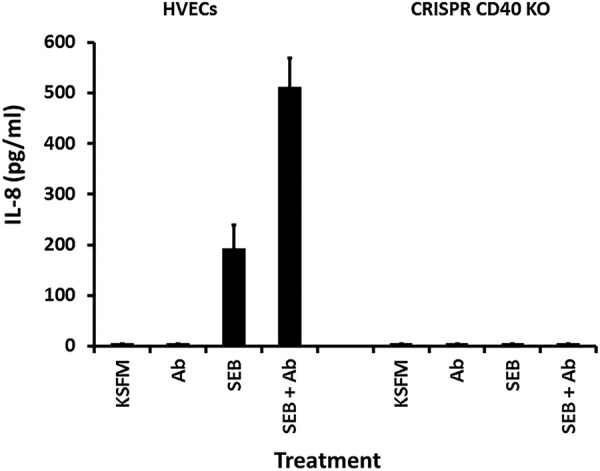
Chemokine (IL-8) production by human vaginal epithelial cells (HVECs) or HVECs knocked out for CD40 after 6-h exposure to SEB (100 µg), SEB (100 µg plus 20 µl of antibodies [Ab] against CD40), 20 µl of antibodies alone, or cells alone in KSFM. Bars indicate standard SD. All responses in the presence of SAg with or without antibodies are significantly greater than HVECs in KSFM alone or KSFM plus antibodies (*P* < 0.001). Additionally, the responses of SAg alone compared to SAg plus antibodies were significantly different (*P* < 0.001). Finally, responses of HVECs to SEB with or without antibodies were greater than HVECs knocked out for CD40 with or without antibodies (*P* < 0.001).

We thus tested whether or not antibodies against CD40 would block SAg stimulation of HVECs to produce chemokines. Surprisingly, stimulation of HVECs individually with the two tested SAgs (TSST-1 and SEB) caused enhanced chemokine production in the presence of the antibodies (Fig. 5 and 6) compared to SAg alone. At matched doses of 10 µg of TSST-1 and SEB (Fig. 5), TSST-1 led to greater IL-8 production by HVECs than did SEB, and the enhanced response was correspondingly greater in the presence of the antibodies against CD40. However, at 100 µg SEB caused similar IL-8 production as 10 µg of TSST-1 (compare Fig. 5 and 6), and the antibodies led to more significant enhancement of IL-8 production (Fig. 6). These data suggested that either the two SAgs were binding to different receptors than the antibodies or the SAgs and antibodies were binding to different parts of CD40.

### CRISPR-Cas9 knockout of CD40 on HVECs eliminates production of chemokines induced by SAgs and SAgs plus antibodies against CD40.

We used CRISPR-Cas9 to knock out CD40 on HVECs. Figure 4 shows flow cytometry after immunofluorescent staining for CD40 versus isotype control of native HVECs (Fig. 4A), HVECs with CD40 knocked out (Fig. 4B), and HVECs with CD40 complemented on a plasmid (Fig. 4C).

**FIG 7:**
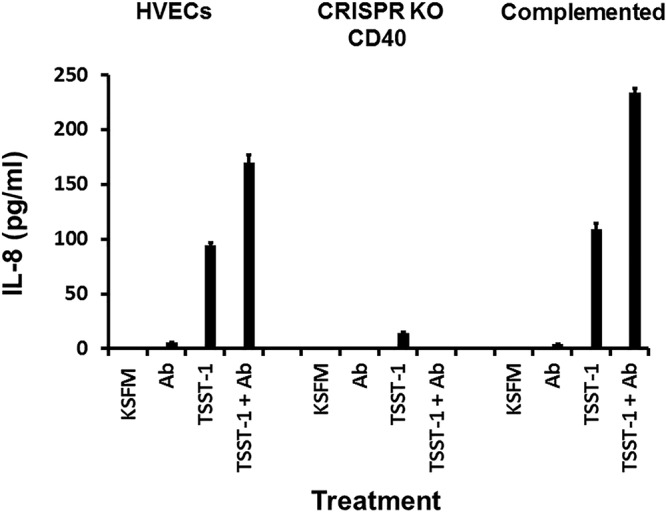
Chemokine (IL-8) production by human vaginal epithelial cells (HVECs), HVECs knocked out for CD40, or HVECs complemented with CD40 after 6-h exposure to TSST-1 (100 µg), TSST-1 (100 µg plus 20 µl of antibodies [Ab] against CD40), 20 µl of antibodies alone, or cells alone in KSFM. Bars indicate SD. All responses in the presence of SAg with or without antibodies are significantly greater than cells in KSFM alone or KSFM plus antibodies (*P* < 0.001), except for HVECs knocked out for CD40. Additionally, the responses of SAg alone compared to SAg plus antibodies were significantly different (*P* < 0.001), except for HVECs knocked out for CD40. Finally, responses of cells to TSST-1 with or without antibodies were greater than HVECs knocked out for CD40 with or without antibodies (*P* < 0.001).

We then tested TSST-1 (Fig. 7) and SEB (Fig. 6) for ability to stimulate chemokine (IL-8) production by HVEC types, native HVECs (both TSST-1 and SEB), HVECs lacking CD40 (both TSST-1 and SEB), and CD40-complemented HVECs (TSST-1). As expected, TSST-1 and SEB stimulated IL-8 production by HVECs, and this response was enhanced in the presence of antibodies against CD40. Both responses (SAg alone and SAg plus antibodies) were completely inhibited in CD40-knockout HVECs. The chemokine response was restored when tested, using TSST-1, on the CD40-complemented HVECs. Collectively, these data make 2 major points: (i) SAgs bound to CD40 to stimulate chemokine production by HVECs, with TSST-1 interacting with greater affinity than SEB or SEC, and (ii) both SAgs and antibodies against CD40 bound to different regions of CD40 to cause amplified chemokine production.

### Wild-type and K121A TSST-1 molecules cause comparable production of IL-8 from HVECs.

**FIG 8:**
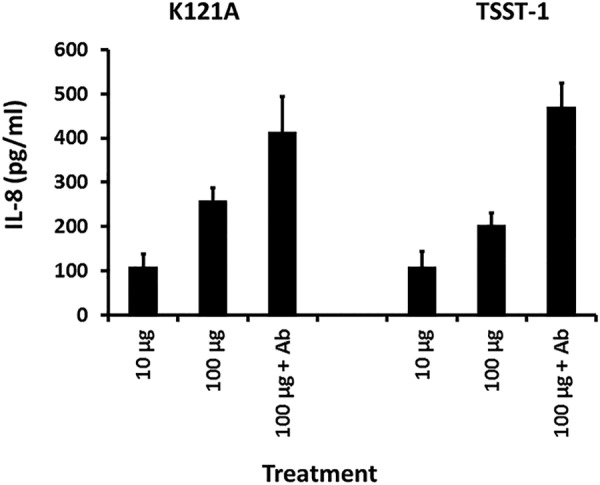
Chemokine (IL-8) production by human vaginal epithelial cells after 6-h exposure in KSFM to the K121A mutant of TSST-1 (10 and 100 µg), K121A mutant (100 µg plus 20 µl of antibodies [Ab] against CD40), TSST-1 wild type (10 and 100 µg), or TSST-1 (100 µg plus 20 µl of antibodies [Ab] against CD40). Bars indicate SD. All responses in the presence of SAg with or without antibodies when matched for doses are not significantly different.

Because the K121A mutant retained full ability to cause TSS as applied intravaginally in rabbits, we expected the mutant would retain the ability to stimulate chemokine production from HVECs comparably to wild-type TSST-1. This was tested (Fig. 8). The wild type and K121A TSST-1 mutant stimulated comparable chemokine (IL-8) production at comparable doses. Additionally, the response of both was enhanced in the presence of antibodies against CD40.

## DISCUSSION

It has been nearly 40 years since menstrual, tampon-associated staphylococcal TSS was identified (29, 30). In the years following, many advances have been made, including identification of the causative toxin in 1981 (14, 15), named TSST-1 in 1984; demonstration that oxygen was the major required factor introduced by tampons to lead to TSST-1 production (31); and demonstration that TSST-1 functioned as a SAg to cause a cytokine storm with resultant TSS (1, 4, 14). Despite these major advances, there have been limited studies to clarify (i) why TSST-1 is the only SAg associated with menstrual TSS, when there are more than 20 other described staphylococcal SAgs, and (ii) how TSST-1 penetrates the multilayered vaginal epithelium. Our study was undertaken to shed light on these unresolved questions.

We have now shown that (i) the submucosal vaginal environment is critical to development of menstrual TSS, (ii) TSST-1 binds to CD40 and the downstream consequence of this binding is epithelial cell production of chemokines that attract other components of the adaptive immune system, and (iii) the specificity of TSST-1 for menstrual TSS is in part dependent on higher-affinity interaction with CD40 than other SAgs, such as SEB and SEC.

A major part of the immune system resides in the submucosa, sites where antigenic exposure would have great likelihood. Our current studies, with use of the K121A mutant of TSST-1, which retains wild-type superantigenicity, show that the submucosal environment vaginally is critical to the ability of TSST-1 to cause menstrual TSS. We have previously shown that superantigenicity and ability of TSST-1 to penetrate vaginal mucosae are separable properties, with data indicating participation of the dodecapeptide region in interaction with the mucosa (11, 12). The human vagina is many cell layers thick, except near the cervix where the epithelium transitions into one cell layer (23). Many studies have shown that certain tampons, notably those of high absorbency, are associated with TSS (29, 30, 32). Additionally, other devices used vaginally, such as diaphragms, contraceptive sponges, and more recently menstrual cups, have been associated with menstrual TSS (1). The shared property of all these devices is introduction of air (oxygen) into a normally quite anaerobic environment. Schlievert and colleagues in multiple studies have shown that oxygen is absolutely required for production of TSST-1, the only toxin associated with menstrual TSS cases (31, 33–36). TSST-1 has also been shown to be produced vaginally with tampon use (37). Once produced, TSST-1 must penetrate the vaginal mucosa. Our studies here suggest that TSST-1 binds to CD40 on vaginal epithelial cells with production of chemokines. The result of chemokine production is proinflammation with further disruption of the mucosal barrier due to harmful inflammation, a characteristic of menstrual TSS seen upon autopsy (38).

Previously, we showed that TSST-1 causes chemokine production from HVECs (18), and we showed that TSST-1 can bind to CD40 (28). However, until now we have not shown the interaction of TSST-1 with CD40 is functional. Interestingly, too, despite numerous trials we have been unable to demonstrate SEB and SEC binding to CD40. The current study suggests that these other two SAgs, which, with TSST-1, are highly associated with nonmenstrual TSS (39, 40), have the ability to bind to CD40 since they (i) stimulate chemokine production, albeit at 10- to 50-fold-higher concentrations than TSST-1, and (ii) the CRISPR-Cas9 knockout HVEC line does not respond to SEB with production of chemokines. It is important to mention that the CRISPR-Cas9 knockout HVECs visibly appear to have the same properties as the wild-type HVECs, those of normal epithelial cell appearance and forming partial tight junctions. The observation that we can restore usual chemokine production in the presence of TSST-1, by addition of CD40 to the knockout cells on a plasmid, strongly indicates there is not a secondary effect on other genes. Collectively, our new findings suggest that TSST-1 (and the K121 mutant of TSST-1) stimulates epithelial cells to produce chemokines locally in the vaginal environment through functional interaction with CD40. This interaction is greater than for SEB or SEC, making TSST-1 the only superantigen with enough activity to disrupt the thick mucosal barrier and cause menstrual TSS. This interaction then attracts and activates submucosal, resident T cells and macrophages to result in TSS.

Unlike menstrual TSS, where the SAgs must penetrate a thick mucosal barrier, postinfluenza TSS, likely the most common form of TSS today, is associated with pulmonary infection where SAgs must traverse at most only one epithelial cell lining lung tissue. This likely explains why TSST-1, SEB, and SEC all may be associated with this form of TSS, noting that even in this setting, TSST-1-induced pulmonary TSS remains more common than SEB- and SEC-associated disease (41).

We do not know the significance, if any, of the enhanced chemokine production by SAgs in the presence of antibodies against CD40. However, we have previously suggested that SAgs, devoid of superantigenicity through mutagenesis, may function as adjuvants by amplifying chemokine production in the presence of other antigens (28). Additionally, such amplification as seen in this study appears to be the result of both SAg and antibody interaction with different parts of the CD40 molecule. We have not mapped those different binding domains. However, we can use this amplification process to study other cell types and their interaction with SAgs.

## MATERIALS AND METHODS

### Superantigens.

TSST-1 was purified from a clone after culture in RN6390 in the presence of erythromycin. RN6390 does not produce detectable SAgs; the strain has the gene only for SE-like X but does not produce detectable protein. SEB was purified from S. aureus strain MNHO, and SEC was purified from strain MNDON. We could not use clones for the SAg production because we are not select agent laboratories with permission to have plasmid clones of wild-type SEs. All SAgs were purified after culture by 80% ethanol precipitation and thin-layer isoelectric focusing. Proteins thus purified were free of contaminants as established by SDS-PAGE and by assays for known exotoxins (proteases, lipase, and cytotoxins) and peptidoglycan and lipopolysaccharide. SAgs were stored lyophilized (TSST-1) or frozen (SEs B and C); the SEs were stored frozen to reduce their biohazards as powders.

### Antibodies and ELISA.

Monoclonal (catalog number MAB6321) and polyclonal anti-human CD40 (catalog number AFG32 affinity-purified goat IgG) antibodies were purchased from R&D Systems and could be used interchangeably. These antibodies were used undiluted at 20 µl/200-µl well of volume. Kits (IL-8 and MIP-3α Quantikine) for chemokine determination were purchased from R&D Systems, Minneapolis, MN, and used exactly as described by the manufacturer.

### Cell lines.

HVECs from a premenopausal woman were described previously (18). HVECs have been observed to function comparably to primary cell lines in expression of surface molecules and in function in tissue culture plates (18). HVECs were cultured in keratinocyte serum-free medium (KSFM) with antibiotics until 24 h before use. At that time, the cells were changed to KSFM without antibiotics. Experiments were performed for 6 h in KSFM without antibiotics. Most experiments were performed by at least two individuals to ensure reproducibility.

### CRISPR-Cas9 knockout cells.

CD40 guide RNA sequences were designed using the CRISPR design tool (crispr.mit.edu) (42) maintained by Feng Zhang (MIT, Cambridge, MA). Two double-stranded guide sequences were generated using the following pairs of oligonucleotides (IDT, Coralville, IA): hCD40 ex1A, CAC CGA GGC AGA CGA ACC ATA GCG and AAA CCG CTA TGG TTC GTC TGC CTC; hCD40 ex1B, CAC CGC CTC TGC AGT GCG TCC TCT and AAA CAG AGG ACG CAC TGC AGA GGC.

The hCD40 ex1A and ex1B oligonucleotides were phosphorylated with T4 polynucleotide kinase and annealed as described previously (43). Phosphorylated double-stranded oligonucleotides were ligated (Quick ligase; NEB) into pX330 Addgene plasmid ID 42230 (44) cut with BbsI and treated with calf intestinal phosphatase. The ligated DNA was used to transform DH5α Escherichia coli (Invitrogen). Plasmids were purified from individual bacterial colonies and sequenced (Iowa Institute of Human Genetics, Genomics Division) to verify proper insertion of the guide sequences. HVECs (4 × 10^6^) were washed and resuspended in 400 µl Opti-MEM (Invitrogen) containing 2.5 µg hCD40 ex1A plasmid, 2.5 µg hCD40 ex1B plasmid, 0.5 µg pEGFP-C1 (Clontech), and 5 µg synthetic double-stranded oligonucleotide (62 bp, random sequence). Cells were transferred to 4-mm cuvettes for electroporation (200 V, 30 ms; BTX electroporator). Five days after electroporation, cells were stained with anti-human CD40 antibody (G28-5) and an appropriate IPTG-labeled secondary antibody. CD40-negative, GFP-expressing cells were sorted into 96-well plates (1 to 5 cells per well) with a FACSAria Fusion flow cytometer (Becton, Dickinson). Clones were expanded and evaluated for CD40 expression by flow cytometry. As a control for various experiments, one of the CD40-deficient HVEC clones was stably transfected (see electroporation conditions, above) with a plasmid encoding hCD40 (45). The plasmid was modified to contain a puromycin resistance cassette in place of the original neomycin cassette. Transfectants were selected with puromycin (2 µg/ml) in bulk culture. Flow cytometry was used to verify CD40 expression by the resulting line.

### Rabbit assays.

TSST-1 and the K121A mutant of TSST-1 were tested *in vivo* using two rabbit models. These models test the ability of SAgs to synergize with lipopolysaccharide (LPS) up to 10^6^-fold, through acceleration of cytokine release; the animals succumb within 48 h whether the superantigen is administered intravenously (i.v.; systemic) or intravaginally (locally) (12, 46). SAgs, dissolved in phosphate-buffered saline (0.005 M sodium phosphate, pH 7.2, 0.15 M NaCl), were given to young adult American Dutch-belted rabbits (1.0 to 2.0 kg) i.v. (10 to 20 µg/ml) or intravaginally (10 µg/kg). Toxins given i.v. were administered in the marginal ear veins. Intravaginal administrations of SAgs were made through catheters threaded into the vaginas of rabbits after anesthesia with ketamine (25 mg/kg; Phoenix Pharmaceuticals, Inc., St. Joseph, MO) and xylazine (20 mg/kg; Phoenix Pharmaceuticals, Inc.). Toxins were delivered intravaginally in 0.1-ml volumes. For all conditions, LPS (5 μg/kg) isolated from Salmonella enterica serovar Typhimurium was administered i.v. in the marginal ear veins 4 h after the initial superantigen dose, and the rabbits were monitored for 48 h. In agreement with the University of Minnesota IACUC, rabbits that failed to exhibit escape behavior and could not remain upright were considered to have lethal TSS and were euthanized with Beuthanasia D (1 ml/kg; Schering-Plough Animal Health Corp., Union, NJ). We followed an approved University of Minnesota IACUC protocol, in agreement with these requirements.

### Statistics.

Data are reported as means ± standard deviations. For comparison of animal survival, Fisher’s exact probability test was used. For other studies, Student’s *t* test analysis of unpaired evenly distributed data was used to evaluate differences in means. In all studied data, *P* < 0.05 was considered significant.

## References

[1] Spaulding AR, Salgado-Pabón W, Kohler PL, Horswill AR, Leung DY, Schlievert PM 2013 Staphylococcal and streptococcal superantigen exotoxins. Clin Microbiol Rev 26:422–447. doi:10.1128/CMR.00104-12. 23824366 PMC3719495

[2] McCormick JK, Yarwood JM, Schlievert PM 2001 Toxic shock syndrome and bacterial superantigens: an update. Annu Rev Microbiol 55:77–104. doi:10.1146/annurev.micro.55.1.77. 11544350

[3] Dinges MM, Orwin PM, Schlievert PM 2000 Exotoxins of *Staphylococcus aureus*. Clin Microbiol Rev 13:16–34. doi:10.1128/CMR.13.1.16. 10627489 PMC88931

[4] Marrack P, Kappler J 1990 The staphylococcal enterotoxins and their relatives. Science 248:705–711. doi:10.1126/science.2185544. 2185544

[5] Kotzin BL, Leung DY, Kappler J, Marrack P 1993 Superantigens and their potential role in human disease. Adv Immunol 54:99–166. doi:10.1016/S0065-2776(08)60534-9. 8397479

[6] Arad G, Levy R, Hillman D, Kaempfer R 2000 Superantigen antagonist protects against lethal shock and defines a new domain for T-cell activation. Nat Med 6:414–421. doi:10.1038/74672. 10742148

[7] Arad G, Levy R, Nasie I, Hillman D, Rotfogel Z, Barash U, Supper E, Shpilka T, Minis A, Kaempfer R 2011 Binding of superantigen toxins into the CD28 homodimer interface is essential for induction of cytokine genes that mediate lethal shock. PLoS Biol 9:e1001149. doi:10.1371/journal.pbio.1001149. 21931534 PMC3172200

[8] Hamad AR, Marrack P, Kappler JW 1997 Transcytosis of staphylococcal superantigen toxins. J Exp Med 185:1447–1454. doi:10.1084/jem.185.8.1447. 9126925 PMC2196287

[9] Vu BG, Stach CS, Kulhankova K, Salgado-Pabón W, Klingelhutz AJ, Schlievert PM 2015 Chronic superantigen exposure induces systemic inflammation, elevated bloodstream endotoxin, and abnormal glucose tolerance in rabbits: possible role in diabetes. mBio 6:e02554-14. doi:10.1128/mBio.02554-14. 25714716 PMC4358007

[10] Vu BG, Gourronc FA, Bernlohr DA, Schlievert PM, Klingelhutz AJ 2013 Staphylococcal superantigens stimulate immortalized human adipocytes to produce chemokines. PLoS One 8:e77988. doi:10.1371/journal.pone.0077988. 24205055 PMC3813495

[11] Shupp JW, Jett M, Pontzer CH 2002 Identification of a transcytosis epitope on staphylococcal enterotoxins. Infect Immun 70:2178–2186. doi:10.1128/IAI.70.4.2178-2186.2002. 11895985 PMC127880

[12] Schlievert PM, Jablonski LM, Roggiani M, Sadler I, Callantine S, Mitchell DT, Ohlendorf DH, Bohach GA 2000 Pyrogenic toxin superantigen site specificity in toxic shock syndrome and food poisoning in animals. Infect Immun 68:3630–3634. doi:10.1128/IAI.68.6.3630-3634.2000. 10816521 PMC97652

[13] Kushnaryov VM, MacDonald HS, Reiser R, Bergdoll MS 1984 Staphylococcal toxic shock toxin specifically binds to cultured human epithelial cells and is rapidly internalized. Infect Immun 45:566–571. 6469347 10.1128/iai.45.3.566-571.1984 PMC263331

[14] Schlievert PM, Shands KN, Dan BB, Schmid GP, Nishimura RD 1981 Identification and characterization of an exotoxin from *Staphylococcus aureus* associated with toxic-shock syndrome. J Infect Dis 143:509–516. doi:10.1093/infdis/143.4.509. 6972418

[15] Bergdoll MS, Crass BA, Reiser RF, Robbins RN, Davis JP 1981 A new staphylococcal enterotoxin, enterotoxin F, associated with toxic-shock-syndrome *Staphylococcus aureus* isolates. Lancet i:1017–1021. doi:10.1016/S0140-6736(81)92186-3. 6112412

[16] Li H, Llera A, Tsuchiya D, Leder L, Ysern X, Schlievert PM, Karjalainen K, Mariuzza RA 1998 Three-dimensional structure of the complex between a T cell receptor beta chain and the superantigen staphylococcal enterotoxin B. Immunity 9:807–816. doi:10.1016/S1074-7613(00)80646-9. 9881971

[17] Fields BA, Malchiodi EL, Li H, Ysern X, Stauffacher CV, Schlievert PM, Karjalainen K, Mariuzza RA 1996 Crystal structure of a T-cell receptor beta-chain complexed with a superantigen. Nature 384:188–192. doi:10.1038/384188a0. 8906797

[18] Peterson ML, Ault K, Kremer MJ, Klingelhutz AJ, Davis CC, Squier CA, Schlievert PM 2005 The innate immune system is activated by stimulation of vaginal epithelial cells with *Staphylococcus aureus* and toxic shock syndrome toxin 1. Infect Immun 73:2164–2174. doi:10.1128/IAI.73.4.2164-2174.2005. 15784559 PMC1087460

[19] Brosnahan AJ, Schaefers MM, Amundson WH, Mantz MJ, Squier CA, Peterson ML, Schlievert PM 2008 Novel toxic shock syndrome toxin-1 amino acids required for biological activity. Biochemistry 47:12995–13003. doi:10.1021/bi801468w. 19012411 PMC2645936

[20] Brosnahan AJ, Schlievert PM 2011 Gram-positive bacterial superantigen outside-in signaling causes toxic shock syndrome. FEBS J 278:4649–4667. doi:10.1111/j.1742-4658.2011.08151.x. 21535475 PMC3165073

[21] Brosnahan AJ, Mantz MJ, Squier CA, Peterson ML, Schlievert PM 2009 Cytolysins augment superantigen penetration of stratified mucosa. J Immunol 182:2364–2373. doi:10.4049/jimmunol.0803283. 19201891 PMC2805182

[22] Davis CC, Kremer MJ, Schlievert PM, Squier CA 2003 Penetration of toxic shock syndrome toxin-1 across porcine vaginal mucosa ex vivo: permeability characteristics, toxin distribution, and tissue damage. Am J Obstet Gynecol 189:1785–1791. doi:10.1016/S0002-9378(03)00873-1. 14710116

[23] Squier CA, Mantz MJ, Schlievert PM, Davis CC 2008 Porcine vagina ex vivo as a model for studying permeability and pathogenesis in mucosa. J Pharm Sci 97:9–21. doi:10.1002/jps.21077. 17721937

[24] Anand SX, Viles-Gonzalez JF, Badimon JJ, Cavusoglu E, Marmur JD 2003 Membrane-associated CD40L and sCD40L in atherothrombotic disease. Thromb Haemost 90:377–384. doi:10.1160/TH03-05-0268. 12958605

[25] Li Q, Estes JD, Schlievert PM, Duan L, Brosnahan AJ, Southern PJ, Reilly CS, Peterson ML, Schultz-Darken N, Brunner KG, Nephew KR, Pambuccian S, Lifson JD, Carlis JV, Haase AT 2009 Glycerol monolaurate prevents mucosal SIV transmission. Nature 458:1034–1038. doi:10.1038/nature07831. 19262509 PMC2785041

[26] Haase AT, Rakasz E, Schultz-Darken N, Nephew K, Weisgrau KL, Reilly CS, Li Q, Southern PJ, Rothenberger M, Peterson ML, Schlievert PM 2015 Glycerol monolaurate microbicide protection against repeat high-dose SIV vaginal challenge. PLoS One 10:e0129465. doi:10.1371/journal.pone.0129465. 26057743 PMC4461171

[27] Schlievert PM, Peterson ML 2012 Glycerol monolaurate antibacterial activity in broth and biofilm cultures. PLoS One 7:e40350. doi:10.1371/journal.pone.0040350. 22808139 PMC3394780

[28] Spaulding AR, Lin YC, Merriman JA, Brosnahan AJ, Peterson ML, Schlievert PM 2012 Immunity to *Staphylococcus aureus* secreted proteins protects rabbits from serious illnesses. Vaccine 30:5099–5109. doi:10.1016/j.vaccine.2012.05.067. 22691432 PMC3397198

[29] Shands KN, Schmid GP, Dan BB, Blum D, Guidotti RJ, Hargrett NT, Anderson RL, Hill DL, Broome CV, Band JD, Fraser DW 1980 Toxic-shock syndrome in menstruating women: association with tampon use. N Engl J Med 303:1436–1442. doi:10.1056/NEJM198012183032502. 7432402

[30] Davis JP, Chesney PJ, Wand PJ, LaVenture M 1980 Toxic-shock syndrome: epidemiologic features, recurrence, risk factors, and prevention. N Engl J Med 303:1429–1435. doi:10.1056/NEJM198012183032501. 7432401

[31] Schlievert PM, Blomster DA 1983 Production of staphylococcal pyrogenic exotoxin type C: influence of physical and chemical factors. J Infect Dis 147:236–242. doi:10.1093/infdis/147.2.236. 6827140

[32] Osterholm MT, Davis JP, Gibson RW, Mandel JS, Wintermeyer LA, Helms CM, Forfang JC, Rondeau J, Vergeront JM 1982 Tri-state toxic-state syndrome study. I. Epidemiologic findings. J Infect Dis 145:431–440. doi:10.1093/infdis/145.4.431. 7069223

[33] Pragman AA, Yarwood JM, Tripp TJ, Schlievert PM 2004 Characterization of virulence factor regulation by SrrAB, a two-component system in *Staphylococcus aureus*. J Bacteriol 186:2430–2438. doi:10.1128/JB.186.8.2430-2438.2004. 15060046 PMC412142

[34] Yarwood JM, McCormick JK, Schlievert PM 2001 Identification of a novel two-component regulatory system that acts in global regulation of virulence factors of *Staphylococcus aureus*. J Bacteriol 183:1113–1123. doi:10.1128/JB.183.4.1113-1123.2001. 11157922 PMC94983

[35] Yarwood JM, Schlievert PM 2000 Oxygen and carbon dioxide regulation of toxic shock syndrome toxin 1 production by *Staphylococcus aureus* MN8. J Clin Microbiol 38:1797–1803. 10790102 10.1128/jcm.38.5.1797-1803.2000 PMC86591

[36] Hill DR, Brunner ME, Schmitz DC, Davis CC, Flood JA, Schlievert PM, Wang-Weigand SZ, Osborn TW 2005 In vivo assessment of human vaginal oxygen and carbon dioxide levels during and post menses. J Appl Physiol 99:1582–1591. doi:10.1152/japplphysiol.01422.2004. 15932958

[37] Schlievert PM, Nemeth KA, Davis CC, Peterson ML, Jones BE 2010 *Staphylococcus aureus* exotoxins are present in vivo in tampons. Clin Vaccine Immunol 17:722–727. doi:10.1128/CVI.00483-09. 20335433 PMC2863369

[38] Larkin SM, Williams DN, Osterholm MT, Tofte RW, Posalaky Z 1982 Toxic shock syndrome: clinical, laboratory, and pathologic findings in nine fatal cases. Ann Intern Med 96:858–864. doi:10.7326/0003-4819-96-6-858. 7091957

[39] Schlievert PM, Tripp TJ, Peterson ML 2004 Reemergence of staphylococcal toxic shock syndrome in Minneapolis-St. Paul, Minnesota, during the 2000–2003 surveillance period. J Clin Microbiol 42:2875–2876. doi:10.1128/JCM.42.6.2875-2876.2004. 15184497 PMC427823

[40] Schlievert PM, Kim MH 1991 Reporting of toxic shock syndrome *Staphylococcus aureus* in 1982 to 1990. J Infect Dis 164:1245–1246. doi:10.1093/infdis/164.6.1245. 1955734

[41] MacDonald KL, Osterholm MT, Hedberg CW, Schrock CG, Peterson GF, Jentzen JM, Leonard SA, Schlievert PM 1987 Toxic shock syndrome. A newly recognized complication of influenza and influenzalike illness. JAMA 257:1053–1058. doi:10.1001/jama.1987.03390080043027. 3806893

[42] Hsu PD, Scott DA, Weinstein JA, Ran FA, Konermann S, Agarwala V, Li Y, Fine EJ, Wu X, Shalem O, Cradick TJ, Marraffini LA, Bao G, Zhang F 2013 DNA targeting specificity of RNA-guided Cas9 nucleases. Nat Biotechnol 31:827–832. doi:10.1038/nbt.2647. 23873081 PMC3969858

[43] Bauer DE, Canver MC, Orkin SH 2015 Generation of genomic deletions in mammalian cell lines via CRISPR/Cas9. J Vis Exp (95):e52118. doi:10.3791/52118. 25549070 PMC4279820

[44] Ran FA, Hsu PD, Wright J, Agarwala V, Scott DA, Zhang F 2013 Genome engineering using the CRISPR-Cas9 system. Nat Protoc 8:2281–2308. doi:10.1038/nprot.2013.143. 24157548 PMC3969860

[45] Hostager BS, Hsing Y, Harms DE, Bishop GA 1996 Different CD40-mediated signaling events require distinct CD40 structural features. J Immunol 157:1047–1053. 8757608

[46] Schlievert PM 1982 Enhancement of host susceptibility to lethal endotoxin shock by staphylococcal pyrogenic exotoxin type C. Infect Immun 36:123–128. 7042568 10.1128/iai.36.1.123-128.1982 PMC351193

